# Supplementary material for: Are carbon emissions trading and green financial instruments synergistic? -Comprehensive quantitative research based on content analysis
Source: PLoS One. 2024 Mar 7;19(3):e0298601. doi: 10.1371/journal.pone.0298601 (PMC10919601; doi:10.1371/journal.pone.0298601)
Supplement: S1 Data — (ZIP) [file pone.0298601.s001.zip › Data/地级市Z_F工作报告.zip!/┴╔─■/┼╠╜⌡╩╨/└┤╫╘╣1⁄2╓┌║┼í╛┬φ┐╦╩2╛▌═°í┐_2012.docx]

各位代表：

现在，我代表市人民政府，向大会报告工作，请予审议，并请市政协各位委员和列席同志提出意见。

一、2011年工作回顾

刚刚过去的一年，在市委的正确领导下，市政府牢牢抓住科学发展这个第一要务，以项目为中心、以改革开放为动力、以服务为保障、以民生为根本，团结带领全市人民，努力拼搏，真抓实干，圆满完成市六届人大二次会议确定的各项任务。

——预计地区生产总值1140亿元，增长15%；

——财政一般预算收入112.3亿元，增长39.3%；

——固定资产投资1050亿元，增长35%；

——社会消费品零售总额217亿元，增长17.5%；

——外贸出口5.3亿美元，增长44%。

在这里我要特别报告的是，全市实际利用外资达到20亿美元，总量全省第三，增速全省第一。其中，兴隆台区突破6亿美元，增长378%，总量全省城区第七； 大洼县达到3.8亿美元，增长154%，总量全省县域第一；盘山县达到2.5亿美元，增长66.3%；双台子区达到2亿美元，增长32.9%。

今天，我们可以自豪地说：经过全市人民的共同奋斗，我们胜利实现了“十二五”的高起点开局，盘锦的各项工作走在了全省前列，一个充满生机与希望的滨海新盘锦正阔步向前！

（一）发展思路更加清晰。按照市第六次党代会的决策部署，我们进一步明确了发展思路、战略目标等一系列根本问题。

1.发展思路上，明确提出跳出盘锦思考盘锦、谋划盘锦，用盘锦的资源，用辽宁的资源，用全国乃至世界的资源，建设盘锦。

2.战略目标上，明确提出建设更具实力、活力和竞争力的滨海新盘锦，让盘锦成为人口达到300万的工业城市、旅游城市、港口城市、宜居城市，综合实力进入辽宁第一集团。

3.产业发展上，明确提出：

——工业要建设世界级石油化工和装备制造基地；

——农业要建设东北重要的农产品深加工基地；

——服务业要建设国内外知名的旅游目的地、区域性的购物中心和环境优美的生态宜居城市。

4.空间布局上，规划了“六大工业园区”、“三大农业平台”、“三大旅游载体”和“五个生态新城”。

——依托“油、化”、港口的优势，建设六大工业园区，即：辽滨沿海经济区产业园、辽宁北方塑料新材料产业园、大洼临港经济区和盘锦精细化工（塑料）产业园、石油天然气装备制造产业园、石油化工产业园；

——依托特色农业、区位交通的优势，建设三大农业平台，即：辽宁盘锦（高升）现代农业综合示范区、辽宁（大洼）现代农机产业园、盘锦（大洼）临港农产品加工及物流园；

——依托芦苇湿地、红海滩、温泉、大海的优势，建设三大旅游载体，即：辽河口生态经济区、红海滩湿地旅游度假区、大洼温泉新城；

——依托生态环境的优势，建设五个生态新城，即：兴隆台生态新城、辽河生态新城、大洼温泉新城、盘山生态新城和辽滨生态水城。

（二）大项目战略取得重大突破。一批具有发动机效应的重大项目纷纷落地。全市亿元以上项目引进182个，其中10亿元以上项目达到66个。

（1）商贸地产大项目21个，主要有：

——总投资100亿元的上海绿地城市综合体；

——总投资100亿元的香港东方银座中心城；

——总投资100亿元的荣盛城市综合体；

——总投资100亿元的珠海华发城市综合体；

——总投资50亿元的广东美的城市综合体；

——总投资50亿元的香港佳兆业城市综合体；

——总投资50亿元的苏宁广场等。

（2）温泉、旅游大项目16个，主要有：

——总投资100亿元的广东碧桂园旅游综合体；

——总投资100亿元的上海三盛温泉小镇；

——总投资75亿元的香港怡海置业水星城；

——总投资50亿元的中南控股旅游会展综合体；

——总投资50亿元的锦联经典生活娱乐城；

——总投资20亿元的珠海天沐温泉城等。

特别是万豪、铂尔曼等一批国际著名的五星级酒店已经陆续开工。

（3）工业大项目22个，主要有：

——总投资845亿元的中国兵器精细化工产业园；

——总投资200亿元的辽宁忠旺铝型材生产基地；

——总投资160亿元的台湾长春石化基地；

——总投资30亿元的沈鼓装备制造；

——总投资20亿元的西班牙戴纳索丁苯橡胶；

——总投资10亿元的美国霍尼韦尔聚乙烯蜡；

——总投资100亿元的东方今典总部基地；

——总投资100亿元的厦门达嘉总部基地等。

（4）农业产业化大项目7个，主要有：

——总投资18亿元的益海嘉里物流及粮油加工；

——总投资20亿元的江西正邦生猪养殖加工；

——总投资18亿元的天津宝迪科技食品工业园；

——总投资12亿元的辽宁禾丰饲料及肉鸡加工；

——总投资11亿元的浙江森禾花卉产业园等。

这些大企业、大项目，为盘锦未来发展奠定了坚实的基础，用大项目推动盘锦大发展已经形成共识并深入人心。

（三）三次产业竞相发展。

1.工业经济加快壮大。大力实施工业五项工程，规模以上工业增加值增长16.5%、增速全省第三，石化及精细化工产业集群销售收入全省第一。全力支持辽河 油田、北方华锦、长城钻探、辽河石化加快发展，大企业中坚作用更加突出。以六大工业园区支撑产业集群加快成长，北沥百万吨沥青扩建、中润聚醚醚酮、宝来石 化、凯特抽油机等项目投产运营，中国兵器精细化工产业园项目正式启动，我市成为国家级化工新材料产业示范基地。我国第一台具有完全自主知识产权的CP- 300自升式钻井平台成功下水，科技创新和新兴产业形成新的优势，高新技术产品增加值增长30%。国有、外资和民营经济齐头并进。

2.现代服务业蓬勃发展。以旅游业拉动服务业发展取得明显成效，服务业投资占全部投资比重提高了7个百分点。全面启动建设三大旅游载体，成功举办国际湿地 旅游周，生态旅游持续火爆，国内外游客达到303万人。大商新玛特、麦凯乐和居然之家等商业购物中心建成开业，红星美凯龙、鹏欣水游城、佳兆业城市综合体 等项目加快建设，兴隆台中央商务区成为我省首批省级服务业集聚区。全省56个城区综合考评，兴隆台区跃居第6，双台子区前进到14名。

3.现代农业迈出新步伐。三大农业平台建设开端良好，盘锦现代农业综合示范区成为辽宁唯一的省级农业综合示范区。农业生产喜获丰收，盘山县成为辽宁“一县 一业”示范县，大洼县荣获全国粮食生产先进县。盘锦大米、河蟹品牌价值不断提升，带动农民人均增收2000元以上。全省44个县综合考评，大洼县跃居第 5，盘山县跨入全省前10。两县两区均受到省政府嘉奖。

（四）城乡建设发生巨大变化。

1.生态城市建设全面展开。以高标准规划引领城市建设，5个生态新城呈现一片大开发、大建设的火热场面，盘锦新的地标——高268米佳兆业万豪酒店开工建 设，恒大华府、东方银座、利港新城、中珠新城、香堤荣府、水榭春城、辽河左岸等一批设计一流的精品建筑拔地而起，城市变得更加美丽，更加现代。县城、新市 镇和农民新型社区建设步伐加快，城乡一体化稳步推进。现在，北京、吉林、黑龙江以及周边地区的人，开始看好盘锦，投资盘锦，迁居盘锦。

2.基础设施建设取得重大进展。38项重点道路交通工程全面推进。高速公路盘锦南站、中华路、西外环、庄林线、惠宾大街、辽河路等新改建工程建成通车，阜 盘高速、盘营客专加快建设，连接东西、畅通南北的大交通体系基本形成。大伙房输水、电网改造、污水和垃圾处理等工程全面推进，全民健身中心投入使用，市中 心医院、老干部活动中心主体完工，城市公共设施进一步完善。

3.生态环境建设显著加强。全力推进绿化工程，造林绿化4.6万亩，辽河生态公园、向海大道、世纪广场等成为城市新的景观，我市荣获省造林绿化先进市。强 化节能减排，主要污染物排放实现控制目标。以“创城”为契机，对重点广场、水系、街道等进行了高水平的综合整治，启动了“金廊”、“银带”建设工程。建设 森林城市、花园城市、宜居城市的目标向前迈出了坚实的一步。

（五）辽滨沿海经济区建设步伐加快。

——港口建设取得实质性进展，6个5万吨专业码头及配套工程快速推进，疏港铁路全线铺轨，以港兴区、区港联动的良好态势开始形成。

——重大项目加快集聚，长春石化、忠旺铝材、合力叉车等一批国内外知名企业相继落户，实际利用外资达到6亿美元。

——城市功能加快形成，盘锦大学、奥体中心、盛京医院分院等加快建设，路网、水网、绿网等工程取得重要进展。

——体制机制创新加快推进，新区发展活力明显增强，各项经济指标在沿海42个重点园区中名列前茅。

（六）民生工程全面落实。

——城乡居民收入大幅提高，农民人均纯收入增长20.5%，达到11750元；城市居民人均可支配收入增长15%，达到24000元。

——新增实名制就业6.9万人，零就业家庭动态为零，城镇登记失业率为2.67%，继续低于全省平均水平。

——社会保障体系更加完善，企业退休人员基本养老金等提标工作全面完成，城乡居民基本养老保险启动并纳入国家试点。

——社会救助工作走在全省前列，全年投入低保救助金1.15亿元。我们做到了不让一个学生因贫失学。

——落实保障性安居工程49797套,其中棚户区改造37765套、农垦危房改造7712套、保障性住房4320套。

——新建标准化幼儿园15所，素质教育被列为国家教育试点项目，我市与沈阳化工大学等三所高校联合办学正式启动，开启了盘锦本科教育的新篇章。

——基层医疗卫生机构实现药物网上集中招标采购、统一配送和零差率销售。在全省率先实现新农合“一卡通”即时报结，最高报销限额由4万元提高到10.5万元。

——文化事业全面发展，乡镇文化站、农家书屋等公共文化设施建设扎实推进，群众文化生活更加丰富多彩。成功举办第六届全民运动会。

——城乡客运公交化进程加快，更新公交车辆108台。新建改建农村公路93条。小街小巷改造步伐加快，消灭了城区“摸黑路”。

——着力加强社会治安、安全生产、食品药品安全、应急救援、人民调解、法律援助等工作，信访工作目标考核连续7年全省第一，社会更加和谐稳定。

（七）体制机制创新取得新突破。

1.创新行政管理。大力简政放权，连续下放了136项市级行政审批管理权限，各县区、经济区和园区焕发出蓬勃活力。

2.创新招商机制。在全市组建工业、农业、旅游、商贸、地产和外资六大专业招商系统，成立13个市直招商局，全市大招商、招大商的态势强劲。

3.创新考评机制。建立以项目为核心的考评体系，人人有压力，人人有动力，“全市抓发展、重点抓项目”的氛围达到了空前的高度。

4.创新融资体系。债券发行、BT、BOT等市场化融资取得丰硕成果。全年新增贷款240亿元，增速全省第一。辽宁鼎旭在美国成功上市，我市企业上市工作实现了零的突破。

5.创新土地管理。创造性地推进增减挂钩、存量挖潜等工作，解决建设用地31平方公里，土地工作取得重大进展。

（八）软环境进一步优化。

——制定出台了“对外开放”、“工业化和工业新型化”、“人才引进和户籍制度改革”等一系列力度空前的政策措施，盘锦成为辽宁新的政策高地。

——强化“马上就办、马上办好”的作风，各级政府机关主动服务意识明显增强，全市尊商、爱商、护商的氛围更加浓厚。

——实行项目服务责任制，对重大项目，明确一名市长、一名县区长、一名局长，一包到底，保证了重大项目顺利实施。

——开展项目服务督查，定期邀请人大代表、政协委员、企业家代表对政府职能部门进行测评，全市行政服务质量和办事效率大幅提高。

今天的盘锦，已经成为国内外客商高度关注的投资热土。

（九）民主法制建设不断加强。

市政府及组成部门自觉接受市人大及其常委会法律监督、工作监督和市政协民主监督，坚持重大事项向人大及其常委会报告、向政协通报制度，办理人大代表建议 125件，政协提案169件。大力推进政务公开，广泛接受社会监督。坚持依法行政、从严治政，规范权力运行，强化行政监察和审计监督，惩治和预防腐败体系 进一步完善。

国防、人防和民兵预备役建设进一步加强，双拥工作扎实推进。民族团结进步事业不断前进，人口和计划生育、统计、物价、广播电视、气象、地震、质监、外事侨务、宗教、贸促、供销、住房公积金、档案、妇女儿童、老龄、残疾人等工作实现新的发展。

（十）干部群众发展激情空前高涨。

在市委的正确领导下，全市上下始终满怀发展激情，敢于面对困难，敢于走进矛盾，开拓创新，高效工作，创造了“盘锦速度”，赢得了省委、省政府的高度赞誉。如今，广大干部群众信心更加坚定，思想更加解放，精神更加振奋，你追我赶，干劲十足！

各位代表！回顾一年来的奋斗历程，我们之所以能够战胜一个又一个困难，取得一个又一个成绩，靠的是省委、省政府和市委的正确领导，靠的是市人大及其常委 会、市政协的监督支持，靠的是全市人民的团结奋斗、真抓实干。在此，我代表市政府，向全市人民致以崇高的敬意！向人大代表、政协委员，向各民主党派、工商 联、无党派人士和社会团体，向离退休老领导、老同志，向驻盘人民解放军、武警部队官兵、公安干警，向辽河油田、北方华锦、长城钻探、辽河石化等驻盘单位， 向一切关心支持盘锦发展的海内外朋友，表示衷心感谢！

总结去年的工作，我们也清醒地认识到，盘锦经济社会发展中还面临不少困难和问题。主要是：经济结构性矛盾依然突出，工业投资还需加大力度，生产要素、资源 环境约束加剧，维护社会稳定、创新社会管理任务十分艰巨，政府自身建设有待进一步加强。对此，我们一定高度重视，认真加以解决。

二、2012年工作目标与任务

各位代表！当前，盘锦正处于一个非常关键的发展时期，做好今年工作至关重要。我们面临的形势错综复杂，挑战也异常严峻。我们必须保持头脑清醒，必须找准突破口，发挥好优势，利用好资源，积极进取，迎难而上，始终让盘锦的经济高速增长，继续走在全省前列。

今年政府工作的总体要求是：全面贯彻落实科学发展观，按照市委六届二次全会的工作部署，以“项目年”、 “工业年”为抓手，实施项目拉动、开放牵动、工业振兴、环境提升、人力资源支撑和体制机制创新重大举措，继续保持经济社会又好又快发展，确保“两个不低 于”，实现盘锦新的跨越！

今年的主要预期目标是：地区生产总值增长15%；财政一般预算收入增长20%；固定资产投资增长30%；社会消费品零售总额增长18%；外贸出口增长20%；城市居民人均可支配收入增长13%；农民人均纯收入增长16%。

（一）招商引资和项目建设再上新台阶。把项目作为头等大事，用项目推动经济发展，用项目推动城市建设，用项目推动民生改善。

1.项目引进要实现新突破。要更大力度地“走出去”、“请进来”，组织好赴香港、台湾、“长三角”、“珠三角”等地区的大型招商活动，全力引进一批世界 500强、行业100强和大国企、大民企、大外企。引进亿元以上项目200个，其中10亿元以上项目不低于50个。

2.项目开工要实现新突破。全过程推进、全方位对接、全天候服务，千方百计变“纸上项目”为“落地项目”、“围墙项目”为“建设项目”。新开工亿元以上项目200个，其中10亿元以上50个。

3.利用外资要实现新突破。我们就是要用外资保证招商引资的质量，用外资保证大企业大项目进入盘锦，用外资的到位保证项目落实。力争实际利用外资达到30亿美元，让盘锦开放的热潮一浪高过一浪。

4.软环境建设要实现新突破。全面简化审批程序，不断提高服务质量和效率。牢固树立“企业至上”的理念，不分内外，不分新老，真心实意地支持企业、服务企业，建设企业家“愿意来、留得住、发展好”的最佳投资环境。

（二）大力建设辽滨沿海经济区。今年是辽滨新区建设特别关键的一年，各项工作必须上台阶、出形象。

1.港口建设要有大进展。启动建设30万吨原油码头及航道工程，力争建成6个5万吨专业码头，确保疏港铁路建成通车，推进沈盘铁路加快建设，提升港口通关效率和服务功能。

2.重大项目建设要有大进步。开工建设华锦千万吨炼油及百万吨烯烃项目，推进长春石化、合力叉车、绿城城市综合体等项目加快建设。新开工10亿元以上项目15个，引进世界500强、行业100强及上市企业9家，实际利用外资7亿美元。

3.基础设施建设要有大提升。确保奥体中心、盘锦大学、盛京医院分院、省实验学校分校等项目如期投入使用，推进道路、供水、供电和污水处理、热源等基础工程加快建设，尽快形成城市功能。

4.体制机制创新要有大突破。充分发挥综合配套改革的政策优势，进一步加大改革创新力度，建立快捷高效的服务机制，以服务赢得新的竞争优势。

辽滨是盘锦未来发展的支撑与希望，一定要奋发有为，成为辽宁沿海开发开放的排头兵！

（三）全力推进工业大振兴。把工业振兴作为最重要的战略举措，力争工业投资增长40%。

1.大力支持和培育大企业大集团。

——全力支持和服务辽河油田、北方华锦、长城钻探和辽河石化，原油稳产1000万吨，推进“气化辽宁”工程、中国兵器精细化工产业园、千万吨炼油扩建等重特大项目建设，以龙头企业带动产业发展。

——全力抓好宝来石化、和运合成橡胶、沈鼓装备制造、戴纳索丁苯橡胶，以及辽宁陕汽改装车、航天晨光油田特种车等22个重点项目，培育一批企业集团，促进中小企业集聚。

——全力抓好现有企业运行，支持企业技术改造和转型升级，做大做强企业规模。

2.大力推进工业园区建设。

——高标准编制园区规划，推进项目向园区集中，提升产业丰厚度，建设特色产业集群。石化及精细化工、塑料新材料、石油天然气装备制造、船舶及海洋工程四大产业集群销售收入要达到1800亿元。

——加大财政投入力度，全市财政投入20亿元以上，全面推进园区道路、供水、供电等基础设施建设。

——加大资本运作力度，每个园区至少成立一个注册资本5000万元以上开发建设公司，多渠道筹措资金。

——大力发展总部经济和工业地产，开工建设标准化厂房80万平方米以上。

3.大力推进工业招商。充分发挥市直、县区和园区工业招商局的作用，国内外并举，打赢工业招商攻坚仗。引进世界500强、行业100强及上市企业30家，引进域内资金300亿元，每个园区实际利用外资至少1亿美元。

4.大力推进科技创新。

——加快建设研发中心，新建省级以上研发中心、企业技术中心5个，公共研发和公共检测平台7个。

——加快企业并购，力争并购科技型企业5户。

——高水平举办国际石油天然气装备博览会。

——加快建设科技企业孵化器，引进和培育一批高新技术企业，以技术创新带动新兴产业发展。

（四）加快发展以旅游业为龙头的现代服务业。把旅游业作为发动机产业，用旅游业的大发展推动现代服务业和城市建设跃上一个新高度。

1.建设国内外知名的旅游目的地。

——全力抓好碧桂园旅游综合体、三盛温泉小镇、天沐温泉城、海泉湾温泉度假中心等项目，加快引进港中旅等大企业，让三大旅游载体建设上台阶、上水平。

——加快建设佳兆业万豪酒店、东方银座铂尔曼酒店、美的万豪公寓式酒店等项目，提升接待能力和服务水平。

——推进中南控股会展中心、左岸文化会展中心、厦门达嘉会展中心等项目建设，发展会展经济、会议经济。

——加快旅游资源整合，开发一批旅游精品项目，办好国际湿地旅游周、湿地风光摄影大赛等活动。

——加大盘锦对外宣传力度，在中央电视台等各大主流媒体宣传盘锦，把盘锦推向全国、推向世界。

2.建设区域性的购物中心。用大企业大项目推动商贸服务业加快发展，下决心再引进一批投资50亿元以上的商贸项目，同时全力抓好佳兆业城市综合体、苏宁广 场、广厦时代城、红星美凯龙等33个重点项目建设，每个县区都要尽快形成一个有规模、有水平的现代服务业集聚区，让盘锦成为区域性的购物中心。

3．建设地域特色鲜明的文化产业基地。全力引进一批有实力、有影响力的文化龙头企业，抓好辽河文化产业园、辽河碑林、田庄台古镇等资源整合与改造建设，挖 掘和提升辽河文化、湿地文化、石油文化、生态农业文化。抓好酷贝拉少儿体验中心、锦联经典生活娱乐城等项目，推进文化产业集群式发展。

（五）大力发展现代农业。进一步加大农业投入，以壮大产业、富裕农民为重点，推进农业大发展。

——加大农业对外开放力度，推进农业产业化，引进10亿元以上农业产业化项目5个。

——建设三大农业平台，突出抓好六和肉鸭、禾丰牧业、正邦生猪、宝迪食品等项目，争创国家级现代农业示范区。

——提升盘锦大米品牌，推进百万亩优质水稻工程，提高大米产业化经营水平。

——实施百万亩河蟹基地工程，重点搞好大河蟹养殖，把胡家河蟹市场建设成为全国最大的河蟹集散地。

——提高农业组织化程度，扩大农业保险范围。新增农民专业合作社100个，土地承包经营权流转面积达到30万亩。

——加强农田水利等基础设施建设，推进百万亩水稻全程机械化，提高农业综合开发和农机装备水平，用科技推动农业加快发展。

（六）建设环境优美的生态宜居城市。

1.加大力度推进城市建设与改造。

——高标准编制实施城市规划，5个生态新城都要独具特色，每座建筑都要成为精品，让绿色、水系和生态成为盘锦城市建设的标志。

——加大新城建设和旧城改造力度，用香港恒大、上海绿地、珠海华发、广东碧桂园这样的大企业，提升城市建设档次与水平，推进房地产业健康发展。

——加快县城、中心镇和旅游小镇建设步伐，两个县城建设要取得突破性进展，稳步推进城乡一体化。

2.加大力度推进基础设施建设。

——道路交通工程。抓紧启动疏港高速公路、辽滨互通立交和滨海大道，加快林丰路大桥、兴辽路、外环路等道路建设，建成阜盘高速、盘营客专。

——“金廊”和“银带”工程。抓好305国道改造和中华路提升，科学布局商贸地产、温泉旅游和工业大项目，打造盘锦的“金廊”和“银带”，让两条大道成为城市的景观大道，拉动发展的黄金大道。

——公共设施工程。竣工市老干部活动中心，推进大伙房输水、城乡垃圾处理等项目建设，配套建设地上地下基础设施，提升公共设施经营、管理和服务水平。

3.加大力度推进生态环境建设。高标准完成辽河流域综合治理，确保太平河、螃蟹沟等6条河流水质达标。加强湿地资源保护。抓好城区、县城、村镇等绿化项目，造林绿化3.7万亩，加快建设森林城市、花园城市。狠抓节能减排，加快淘汰落后产能。

（七）切实保障和改善民生。

做好今年的民生工作，要在巩固原有成果的基础上，继续抓好免费就业培训、零就业家庭动态扶助等就业工作，进一步完善社会保障体系，推进义务教育均衡发展， 提高医疗保障和公共卫生服务水平，发展文化体育事业，认真落实文化惠民、医疗惠民、农村土危房改造等民生工程，使群众得到更多实惠。

重点办好10件关系群众切身利益的民生实事：

（1）加大财政投入，让所有农村中小学校都用上安全可靠的新校车，确保学生安全。

（2）大力推动幼儿园连锁发展，重点抓好农村幼儿园建设，力争年内一镇建成一所标准化中心幼儿园。

（3）建立和完善城乡贫困家庭学生全程资助、全面覆盖的助学体系，绝不让学生因贫失学。

（4）开工建设市民政福利中心，尽快让孤残儿童、老荣军、城市“三无”老人享受到一流服务。

（5）启动实施残疾人社区康复工程，搞好残疾人康复服务，提高残疾人生活质量。

（6）竣工市中心医院，启动市中医院和市妇女儿童医院选址新建工程。

（7）新建盘山天鹅湖、大洼森林广场、双台子兆林、兴隆台金宝、辽滨金雁湖等9座公园，让群众拥有更多休闲健身的好去处。

（8）解决历史遗留的城区居民已入住房屋产权办理问题。

（9）大力推进便民畅通工程，新建和改造维修农村公路200公里、小街小巷15条，方便群众出行。

（10）整合优化客运公交资源，科学布局客运公交线路，规划建设全市公交客运枢纽中心，提高客运公交服务水平。

我们要持之以恒地坚持下去，每年办好一批摸得着、看得见的民生实事，让群众的生活过得越来越好！

（八）千方百计保障要素。

1.做大人力资源规模。把人才和人口引进作为一项刻不容缓的战略性工程，支撑盘锦大发展、快发展。

——大力发展职业教育，从今年开始，全市财政每年安排2亿元以上资金支持院校建设，同时鼓励企业参与投资。今年，盘锦职业技术学院、辽河油田职业技术学院等11所院校建设都要有大的突破。

——全面实施户籍、购房、社会保障等一系列优惠政策，吸引更多的优秀人才，集聚更多的城市人口。

——加大力度引进一批国际学校，今年至少引进4所，让盘锦尽快成为东北一流的国际教育基地。

——巩固发展基础教育，进一步抓好迁建、扩建和新建中小学校项目，推进农村初中进县城办学。

2.做好资金保障。

——鼓励支持金融机构扩大信贷规模，加大力度落实农业发展银行、国家开发银行等授信贷款项目，让更多的资金流向盘锦。

——继续抓好企业上市，加大基金利用力度，推进债券发行、BT、BOT等市场化运作方式，吸引和带动更多的社会资本。

——建立充满活力的金融服务体系，规范提升融资担保机构，鼓励发展小额贷款公司，加大中小微企业贷款扶持力度。

3.做强土地保障。

——向增减挂钩要土地，在改善农民生活、提高耕地质量的基础上，加大土地置换力度，拓展发展空间。

——向潜在资源要土地，扩大未利用地使用规模。

——向存量要土地，加大城中村、平房区、旧城区和县城的改造力度，盘活土地存量。

——向管理要土地，节约集约用地，严把项目供地标准，提高土地利用率和产出率。

（九）不断加强和创新社会管理。

——创新完善非公经济组织和社会组织管理。搞好流动人口和特殊人群服务管理。推进城乡社区管理创新，提高基层社会管理服务能力。

——巩固和放大“创城”效应，下力量搞好城乡环境综合整治，使城乡秩序更加井然有序，让群众生活更加舒适。

——建立健全城乡一体的社会治安防控、信访工作体系，完善群众利益诉求表达等机制，加强接待和服务群众工作。争创全国综合治理先进市。

——全面加强生产安全、食品药品安全、消防安全和交通安全监管，坚决遏制重特大事故发生。强化应急管理，提高应急处置能力。加强防灾减灾体系建设。

积极支持驻盘部队建设，做好国防动员、国防教育、民兵预备役和人防、海防工作，提高双拥工作水平，巩固军政军民团结。全面做好人口和计划生育、民族宗教、统计、质监、妇女、儿童、老龄等工作。

（十）继续加大政府自身建设力度。

我们要依法行政。自觉接受人大及其常委会依法监督和政协民主监督。推进科学民主决策，深化政务公开，确保各项工作部署更加符合实际，符合群众利益。

我们要讲求诚信。大力加强信用盘锦建设，各级政府要当标兵、做表率，对企业和群众作出的承诺要说到做到，提高政府公信力。

我们要真抓实干。强化工作抓实，不搞花架子，不搞形象工程，为盘锦发展和人民福祉，办真事、办实事、办好事。

我们要高效工作。大力倡导事不过夜、事无巨细、事必躬亲的作风，只争朝夕，杜绝拖沓，雷厉风行，不达目的决不罢休。

我们要敢于担当。树立强烈的责任意识，敢于面对挑战，敢于走进矛盾,解放思想，打破常规，用改革的办法破解难题，创造性地推进工作。

我们要廉洁从政。强化对行政权力的制约和监督，坚决查处违纪违法案件。坚持厉行节约，反对铺张浪费，建设勤政廉政的服务型政府。

各位代表、同志们，盘锦已经进入大发展、快发展的新阶段。加快崛起，强市富民，是全市人民的期盼。在新的一年里，我们一定不负人民的重托与厚望，在市委的 正确领导下，一时也不放松，一刻也不停留，凝心聚力，激情拼搏，努力创造无愧于时代、无愧于人民的新业绩。我们坚信，盘锦人民一定会更加幸福，盘锦的明天 一定会更加美好！
